# Clinicopathologic factors that influence prognosis and survival outcomes in men with metastatic castration‐resistant prostate cancer treated with Radium‐223

**DOI:** 10.1002/cam4.4125

**Published:** 2021-07-13

**Authors:** Esmail M. Al‐Ezzi, Husam A. Alqaisi, Marco A. J. Iafolla, Lisa Wang, Srikala S. Sridhar, Adrian G. Sacher, Nazanin Fallah‐Rad, Di M. Jiang, Geoffrey A. Watson, Charles N. Catton, Padraig R. Warde, Rob J. Hamilton, Neil E. Fleshner, Alexandre R. Zlotta, Aaron R. Hansen

**Affiliations:** ^1^ Division of Medical Oncology and Hematology Princess Margaret Cancer Centre Toronto ON Canada; ^2^ Department of Radiation Oncology Princess Margaret Cancer Centre Toronto ON Canada; ^3^ Division of Urologic Oncology Princess Margaret Cancer Centre Toronto ON Canada; ^4^ Department of Biostatistics Princess Margaret Cancer Centre Toronto ON Canada

**Keywords:** clinical management, neoplasms, prognostic factors, prostate cancer, risk model, survival

## Abstract

**Background:**

In men with metastatic castration‐resistant prostate cancer (mCRPC) with primarily bone metastases, radium‐223 (^223^Ra) improves overall survival (OS). However, the selection of ^223^Ra is not guided by specific validated clinicopathologic factors, and thus outcomes are heterogeneous.

**Patients and methods:**

This retrospective survival analysis was performed in men with mCRPC treated with ^223^Ra at our cancer center. Demographics and disease characteristics were collected. OS was calculated using the Kaplan–Meier method (log‐rank). The potential prognostic factors were determined using both univariable (UVA) and multivariable analysis (MVA) (Cox‐regression) methods.

**Results:**

In total, 150 patients with a median age of 74 years (52–93) received ^223^Ra between May 2015 and July 2018, and 58% had 6–20 bone metastases. Ninety‐four (63%) patients received >4 ^223^Ra doses, and 56 (37%) received ≤4. The following pre‐treatment factors were analyzed (median [range]): eastern cooperative oncology group performance status (ECOG PS), (1 [0–3]); Albumin (ALB), (39 g/L [24–47]); alkaline phosphatase (ALP), (110 U/L [35–1633]); and prostate‐specific antigen (PSA), (49 µg/L [0.83–7238]). The median OS for all patients was 14.5 months (95% CI: 11.2–18). These factors were associated with poor survival outcomes in UVA and MVA: ALB <35 g/L, ALP >150 U/L, ECOG PS 2–3, and PSA >80 µg/L. By assigning one point for each of these factors, a prognostic model was developed, wherein three distinct risk groups were identified: good, 0–1 (*n* = 103); intermediate, 2 (*n* = 30); and poor risk, 3–4 points (*n* = 17). The median OS was 19.4, 10.0, and 3.1 months, respectively (*p *< 0.001).

**Conclusions:**

Pre‐treatment ALB, ALP, ECOG, and PSA, were significantly correlated with OS and could guide treatment selection for men with mCRPC by identifying those who are most or least likely to benefit from ^223^Ra. Validation in an independent dataset is required prior to widespread clinical utilization.

## Graphical abstract

Baseline clinicopathological factors were associated with survival for men with metastatic castration‐resistant prostate cancer who were treated with radium‐233. Our retrospective analysis defined a prognostic model that can be utilized to evaluate the treatment benefit of radium‐223.

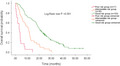

## 1 INTRODUCTION

Prostate cancer is the second leading cause of cancer in men and the fifth most common cause of all cancer deaths globally.^1^ Bone metastases are significant in prostate cancer with approximately 30% of men developing bone metastases in the first 2 years of castration resistance and ultimately 90% of men developing bone metastases over the course of their entire disease.^2^ Skeletal‐related events (SRE) such as pain, fracture, spinal cord compression, hypercalcemia, and bone marrow suppression will occur in over 50% of men with advanced prostate cancer and bone metastases.^3^ Taxane chemotherapy (docetaxel and cabazitaxel), androgen receptor axis‐targeted (ARAT) therapies (abiraterone and enzalutamide), sipuleucel‐T, and radium‐223 (^223^Ra) have demonstrated improved overall survival (OS) metastatic castration‐resistant prostate cancer (mCRPC).^4^, ^5^, ^6^, ^7^, ^8^, ^9^

^223^Ra is a calcium mimetic, alpha‐emitting nuclide, which is taken up in bone metastases due to high bone turnover. The alpha radiation induces double‐strand DNA breaks, causing localized cytotoxicity in bone metastases.^10^ The ALSYMPCA trial was an international, double‐blind, placebo‐controlled phase III trial that enrolled patients with mCRPC and symptomatic bone metastases and randomized them to either ^223^Ra or placebo. This trial reported a statistically significant improvement in median OS in patients treated with ^223^Ra, demonstrating an improvement in OS by 3.6 months (HR 0.70; *p *< 0.0001) and delaying the time to first symptomatic SRE by 5.8 months (HR 0.66, *p *< 0.0001).^9^ Of the men enrolled, 71% had improvement in their pain scores and activity at week 8.^11^ ALSYMPCA data on quality of life (QOL) showed that ^223^Ra increased EQ‐5D utility scores and resulted in a slower decrease in QOL scores as compared to placebo.^12^ Based on the results of this pivotal trial, ^223^Ra was approved for patients with mCRPC with symptomatic bone metastases.^9^, ^13^

Prostate‐specific antigen (PSA) and conventional imaging are routinely used to evaluate treatment response to other treatments, including chemotherapy and ARATs. However, during ^233^Ra treatment, there are no reliable clinical methods to assess response. Hence accurate patient selection at the start of treatment will permit those who are unlikely to benefit from ^233^Ra to avoid this agent while treating those who have the highest chance of benefit. Identification of prognostic factors would guide treatment decision making, improve patient outcomes, and could better inform future clinical trial design. Our aim was to analyze the survival outcomes of men with mCRPC to the bone who were treated with ^223^Ra and to determine the prognostic factors that may affect their OS.

## 2 PATIENTS AND METHODS

### 2.1 Patients and data collection

Men with mCRPC treated with ^223^Ra at our cancer center from May 2015 to July 2018 were included in this study to permit sufficient follow‐up. The study received ethics approval from our institutional review board (REB #18‐5545). Demographics and disease characteristics were obtained from the electronic health records. Baseline variables such as eastern cooperative oncology group performance status (ECOG PS), number of bone metastasis on conventional imaging, lymph node (LN) status, serial PSA, lactate dehydrogenase (LDH), hemoglobin (Hb), albumin (ALB), and alkaline phosphatase (ALP) were collected. Each patient received ^223^Ra 50–55 kBq/kg intravenously q 4 weeks for at least one dose to a maximum of six doses. Information on the number of pre‐ and post‐^223^Ra systemic treatments were collected. The use of bone protecting agents (BPA) such as zoledronate and denosumab was noted. PSA doubling time (PSADT) before and during ^223^Ra treatment were calculated using Memorial Sloan Kettering Hospital calculator.^14^ On treatment serial PSA and ALP levels were obtained. Hematological toxicities such as anemia (Hb <100 g/L), neutropenia (ANC <1 × 10^9^ /L), and thrombocytopenia (platelet <100 × 10^9^/L) as well as fatigue were collected. The number of bone metastases post‐ ^223^Ra therapy was also recorded.

### 2.2 Statistical analysis

Summary statistics using the median and ranges were calculated for demographic and prognostic factors. For survival calculations, time from starting ^223^Ra to the event of interest was used for progression‐free survival (PFS) (disease progression) and OS (death from any cause). Survival analysis was performed using the Kaplan–Meier method (log‐rank). Pre‐treatment clinicopathological variables were analyzed for OS associations using a cox proportional hazards model for univariable (UVA) and selected multivariable analysis (MVA). Hazard ratios (HR) with 95% confidence intervals (95% CI) are reported. A four‐variable prognostic score model was proposed based on the MVA results. A time‐dependent receiver operating characteristic curve (ROC) with an area under curve (AUC) was used to assess the prognostic score's significance. All tests used a *p* value of ≤0.05 for significance. IBM SPSS Statistics v26 was used to conduct statistical analyses (IBM; Armonk, NY, USA).

## 3 RESULTS

### 3.1 Patients’ characteristics

In total, 150 mCRPC patients with a median age of 74 years (52–93) received ^223^Ra between May 2015 and July 2018. Patient and disease characteristics are detailed in (Table 1). Twenty‐one patients (14%) had <6 bone metastases, 87 (58%) had 6–20, and 42 (28%) had > 20. The mean number of ^223^Ra doses received was five (94 patients [63%] received > 4 doses and 56 [37%] received ≤4). The median ECOG PS was 1 with a range (0–3). The presence of LN metastasis, the number of prior lines, and the use of BPAs are detailed in (Table 1). Specifically, prior to ^223^Ra, 65 (43%) patients had received docetaxel, 11 (7%) had received both docetaxel and cabazitaxel, and 62 (41%) had prior ARAT with either enzalutamide or abiraterone. Seventy‐seven patients received BPAs during ^223^Ra (zoledronate 46 [31%] and denosumab 31 [21%]). Pre‐treatment laboratory results such as ALP, Hb, PSA, LDH, ALB, and PSADT were reported (Table 2).

**TABLE 1:** Patients and treatment demographics

Variables	All Patients (*n* = 150)
Median Age (years)	74 (52–93)
Median BMI (kg/m^2^)	27 (15.5–42.1)
ECOG PS 0/1/2/3	13/107/27/3
ECOG PS 0–1/2–3	120(80%)/30(20%)
Bone metastases <6/6–20/>20	21(14%)/87 (58%)/42 (28%)
Mean no. of bone metastasis	12 (6–20)
Presence of LN metastasis	35 (23.3%)
Median no. of previous treatments	2 (0–3)
Docetaxel only	65 (43%)
Docetaxel and Cabazitaxel	11 (7.3%)
Enzalutamide or Abiraterone only	62 (41%)
No. of patients received BPA	77 (51%)
Zoledronic acid/Denosumab	46 (30%)/31 (21%)
Mean no. of ^223^Ra doses	5 (1–6)
≤4 vs. >4 doses	56 (37%)/94 (63%)
No. of patients with hematological toxicity after completion of ^223^Ra^†^	61 (41%)
No. of patients with fatigue after ^223^Ra	75 (50%)
Median no. of post‐ ^223^Ra systemic therapies	1 (0–4)
No. of patients with PSA response during ^223^Ra	27 (18%)
No. of patients with ALK reduction ≥30%	93 (62%)

Abbreviations: ^223^Ra, radium 223; ALK, alkaline phosphatase; BMI, body mass index; BPA, bone protecting agent; ECOG PS, eastern cooperative oncology group performance status; LN, lymph node.

† Hematological toxicities include one of the following: anemia (Hb<100 g/L), neutropenia (ANC<1 × 10^9^/L), and thrombocytopenia (platelet<100 × 10^9^/L).

**TABLE 2:** Pre‐treatment laboratory variables level

Pre‐treatment laboratory variable (*n* = 150)	Median (range)
ALP U/L	110 (35–1633)
Hb g/L	120 (69–160)
PSA µg/L	49 (0.83–7238)
LDH U/L	230 (82–1426)
ALB g/L	39 (24–47)
PSADT(months)	2.4 (−27.2–218.8)

Abbreviations: ALB, albumin; ALP, alkaline phosphatase; Hb, hemoglobin; LDH, lactic dehydrogenase; PSA, prostate‐specific antigen; PSADT, prostate‐specific antigen doubling time.

### 3.2 Clinical outcomes

**FIGURE 1:**
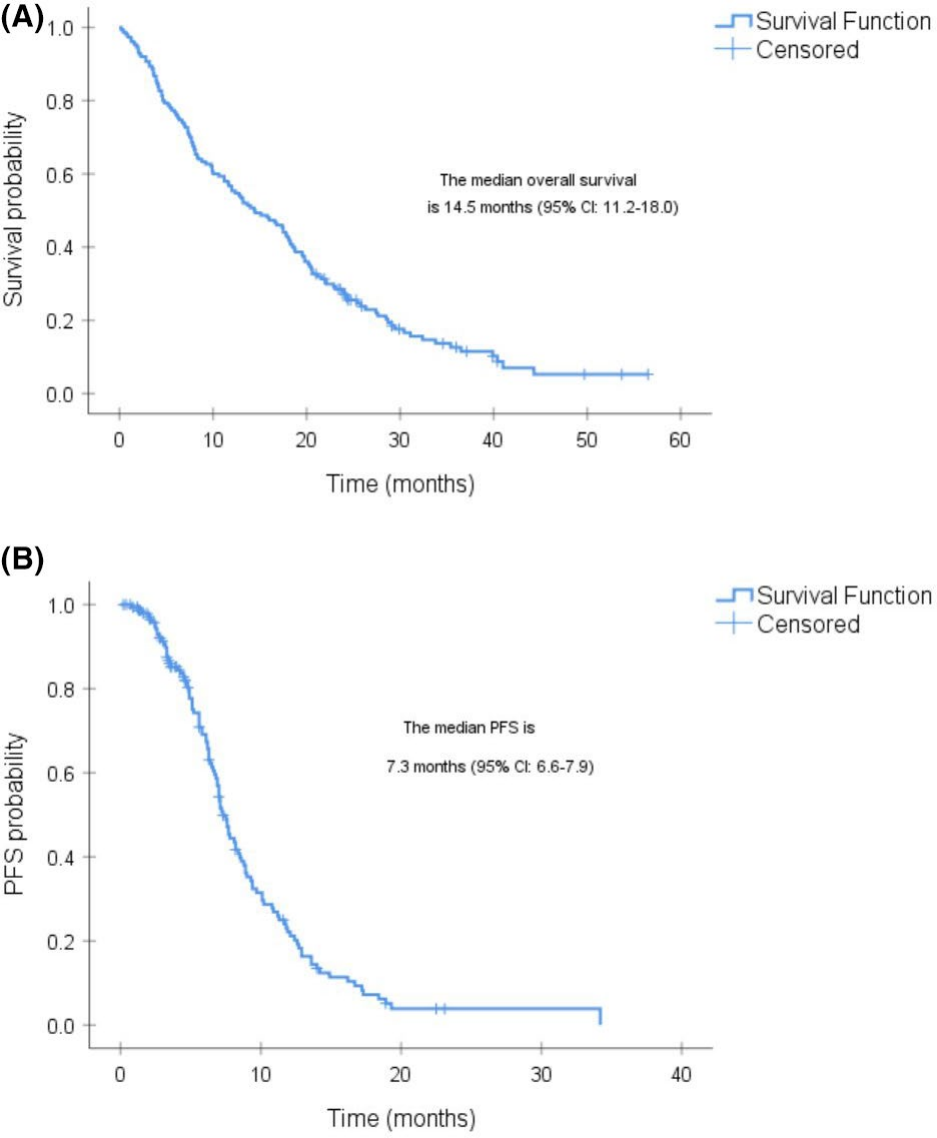
(A) Kaplan–Meier survival curve of the overall survival (OS) of the entire cohort after radium‐223 treatment. (B) Kaplan–Meier survival curve of the progression‐free survival (PFS) of the entire cohort after radium‐223 treatment

A total of 130 men had died, and 110 men had progressed at the time of analysis with a median follow‐up of 39 months (range, 35–45 months). The median OS for all men was 14.5 months (95% CI: 11.2–18.0) (Figure 1A). The median PFS was 7.3 months (95% CI: 6.6–7.9) (Figure 1B).

On UVA, pre‐treatment ALB <35 g/L (HR 2.5, 95% CI: 1.6–4.0; *p *< 0.001), Hb < 120 g/L (HR 2.0, 95% CI:1.4–2.9; *p* < 0.001), ECOG PS 2‐3 (HR 2.4, 95% CI: 1.5–4.0; *p* < 0.001), ALP > 150 U/L (HR 2.2, 95%CI:1.6–3.3; *p *< 0.001), PSA > 80 µg/L (HR 2.0, 95% CI:1.5–3.0; *p *< 0.001), LDH > 220 U/L (HR 1.8, 95% CI: 1.3–2.6, *p* = 0.001), and PSADT< 3 months (HR 1.5, 95%CI:1.0–2.2; *p *= 0.026) were associated with worse OS. On MVA, pre‐treatment albumin <35 g/L (HR 2, 95% CI: 1.2–3.2; *p =* 0.005), ECOG PS 2‐3 (HR 3, 95% CI: 2–4.7; *p* < 0.001), ALP > 150 U/L (HR 1.8, 95%CI:1.3–2.7; *p *= 0.002), and PSA > 80 µg/L (HR 1.7, 95% CI:1.2–2.5; *p =* 0.006) were associated with poor survival outcomes.

On UVA, treatment, and post‐treatment factors, such as >4 ^223^Ra doses (HR 0.19, 95% CI: 0.13–0.28; *p <* 0.001), absence of any hematological toxicity after ^223^Ra completion (HR 0.39, 95% CI: 0.27–0.56; *p <* 0.001), receipt of systemic therapy after ^223^Ra (HR 0.34, 95% CI: 0.24–0.49; *p <* 0.001), and any biochemical PSA response during ^223^Ra therapy (HR 0.53, 95% CI: 0.33–0.63; *p *= 0.01) were associated with better OS. Each of these factors was independently confirmed on MVA , >4 ^223^Ra doses (HR 0.29, 95% CI: 0.19–0.44; *p <* 0.001), absence of any hematological toxicity after ^223^Ra completion (HR 0.5, 95% CI: 0.34–0.69; *p <* 0.001), receipt of systemic therapy after ^223^Ra (HR 0.48, 95% CI: 0.32–0.73; *p <* 0.001), and any biochemical PSA response during ^223^Ra therapy (HR 0.52, 95% CI: 0.32–0.65; *p *= 0.012).

A MVA for those patients who received BPA (zolendronate or denosumab) which included PSA, ALK, ALB, number of cycles, HB, LDH, and baseline PSA doubling time showed HR 0.674 (95%CI: 0.46–0.98; *p* = 0.03) that favored the receipt of BPA.

### 3.3 Prognostic survival model

**FIGURE 2:**
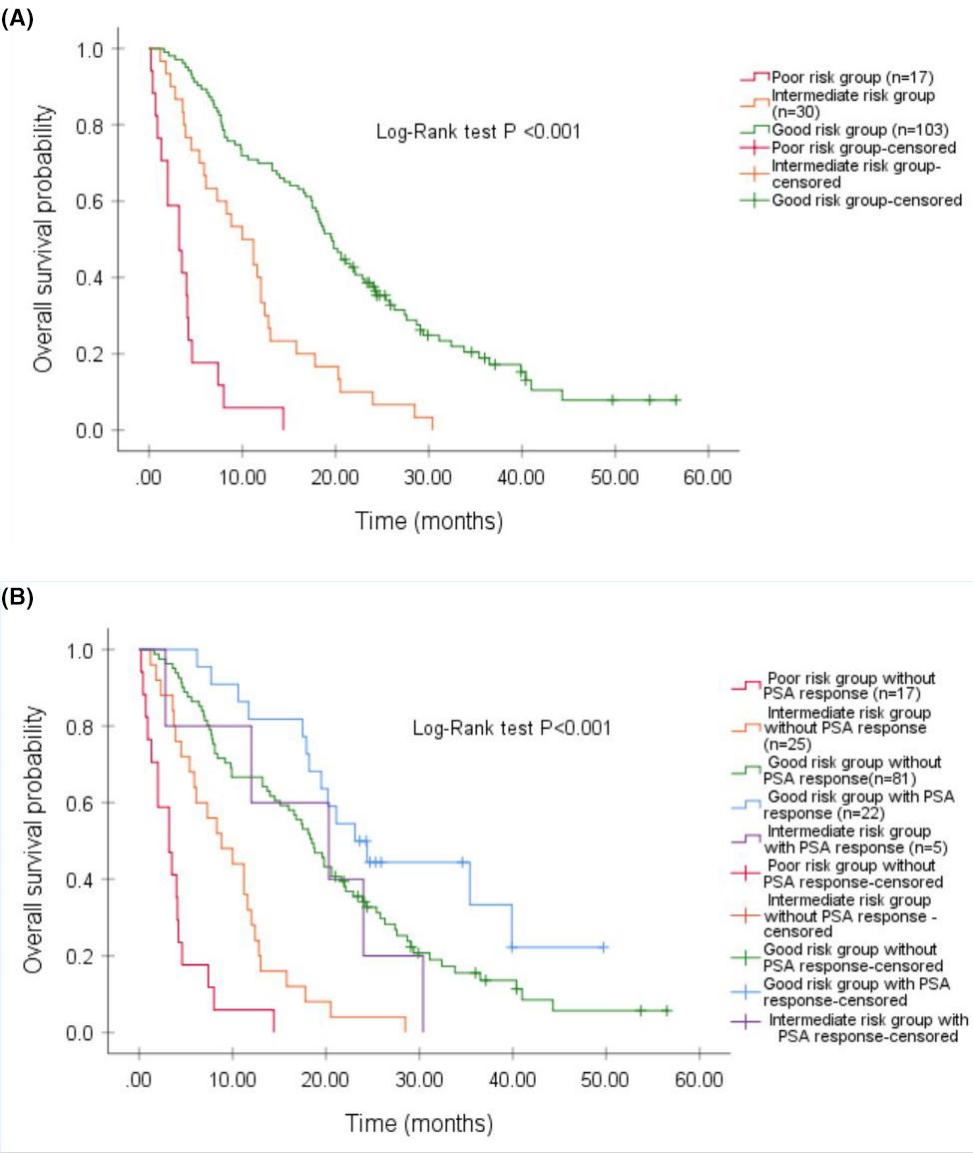
(A) Kaplan–Meier survival curves of the entire cohort after radium‐223 treatment stratified by prognostic risk group. (B) Kaplan–Meier survival curves of the entire cohort after radium‐223 treatment stratified by prognostic risk group and the presence of any PSA response

From a prognostic model based on the four independent, pre‐treatment clinicopathological factors, albumin <35 g/L, ECOG PS 2–3, ALP >150 U/L, and PSA>80 µg/L; three distinct prognostic groups were identified by assigning one point for each aforementioned variable (Table 3). The good, intermediate, and poor risk groups were defined as 0–1 point (*n* = 103), 2 points (*n* = 30), and 3–4 points (*n* = 17), respectively, with a median OS of 19.4 months (95% CI 17.4–21.8), 10.0 months (95% CI 6.1–13.9), and 3.1 months (95% CI 1.2–5.1), respectively; *p* < 0.001 (Figure 2A). Interestingly, patients in the good (*n* = 22) or intermediate risk (*n* = 5) groups who achieved any PSA reduction during ^223^Ra therapy had improved median OS to 23.1 months (95% CI 17.1–29), and 20.0 months (95% CI 2.5–38), respectively; *p *< 0.001 (Figure 2b). The receiver operating curve for the prognostic model score and survival status produced an AUC 0.762, *p *< 0.001. Two patient vignettes were described in Appendix S1.

**TABLE 3:** Four‐variable prognostic score model^†^

Variables	Prognostic score point
Baseline ECOG PS	
0–1	0
2–3	1
Baseline PSA	
≤ 80 µg/L	0
> 80 µg/L	1
**Baseline ALB**	
≥ 35 g/L	0
< 35 g/L	1
**Baseline ALP**	
ALP ≤150 U/L	0
ALP >150 U/L	1

Abbreviations: ALB, albumin; ALP, alkaline phosphatase; ECOG, eastern cooperative oncology group performance status; PSA, prostate‐specific antigen.

† The four‐variable prognostic score model identifies three prognostic groups: good risk, 0 or 1 point; intermediate risk, 2 points; and poor risk, 3 or 4 points.

## 4 DISCUSSION

We identified three different prognostic groups based on a combination of four pre‐treatment clinical and laboratory factors that affected OS in our cohort of 150 patients treated with ^223^Ra during the last 5 years. These factors were ALB less than 35 g/L, ALP above 150 U/L, PSA above 80 µg/L, and ECOG PS score of 2–3. Our analysis identified good, intermediate, and poor risk groups, and these factors may inform the selection of ^223^Ra for men with mCRPC who are most likely to receive a substantial benefit.

This real‐world cohort analyzed in this study had similar outcomes to those reported in the ALSYMPCA trial, where patients treated with ^223^Ra had a median OS of 14.0 months. In addition, ALSYMPCA reported a median OS of 6.2 months in those who received ≤4 doses and 17.9 months with >4 doses. In our cohort the median OS was 5.2 months in those who received ≤4 doses and 20.3 months with >4 doses. Strikingly, our intermediate risk group performed similar to the placebo group in the ALSYMPCA study, but our poor risk group did dramatically worse.

Different factors have demonstrated prognostic value in men with mCRPC. Smaletz et al^15^ reported a baseline prognostic survival model for patients with progressive mCRPC treated with 19 consecutive therapeutic protocols, using some prognostic variables such as age, Karnofsky performance status (KPS), and baseline laboratory values such as PSA, Hb, LDH, ALB, and ALP. On MVA, only KPS, Hb, ALP, ALB, and LDH were significantly associated with OS (*p* < 0.05), however, age and PSA were not.

Low pretreatment ALB is a well‐recognized factor associated with poor survival in cancer patients, including prostate cancer, as reported in a systematic review by Gupta et al.^16^ Interestingly, even at in early stage prostate cancer, low pretreatment ALB can predict a higher pathological T‐stage (T3 or T4) after radical prostatectomy as reported by E Richter et al.^17^ A MVA by Li et al^18^ identified a prognostic nutritional index that included ALB. Elevated pretreatment ALB was a favorable prognostic factor for PFS, cancer‐specific survival, and OS of the metastatic prostate cancer patients.

Bone‐specific prognostic factors, including a history of previous skeletal‐related event^19^, presence of pathologic fracture^20^, urinary N‐telopeptide, and bone‐specific ALP levels^21^, have also demonstrated a prognostic value.

A number of studies have reported factors associated with prognosis to ^223^Ra treatment. Sartor et al^22^ reported an exploratory study from the phase III ALSYMPCA trial in an attempt to correlate baseline patient variables and survival on ^223^Ra. On MVA of these prognostic variables, men who presented with poor baseline ECOG PS, LDH >245 U/L, or ALP >131 U/L were associated with worse survival outcomes. Whereas men on placebo receipt with elevated baseline LDH >266 U/L and ALP >153 U/L were found to be associated with a statistically higher death rate. Notably, PSA was found to have decreased in 27% of ^223^Ra patients, compared with 18% in our cohort. Wongetal et al^23^ evaluated 64 patients with mCRPC who received ^223^Ra based on retrospective analysis and found on MVA, 3 factors prior to ^223^Ra were associated with better OS, which were baseline ALP < 115 U/L, no prior chemotherapy, and ≤ five bone metastases.

Our study corroborated the observation that patients who receive >4 doses of ^223^Ra have better OS compared to those receive ≤4 doses. Parikh et al^24^ reported on prognostic factors from a retrospective study of 189 men treated with ^223^Ra for mCRPC. The median OS of the whole study group was 10.5 months. They defined four factors as prognostic for OS: number of ^223^Ra doses (>4 doses vs. ≤4 doses [HR 0.1; *p* ≤ 0.001]), age>72 years (HR 1.07; *p* = 0.005), neutrophil‐to‐lymphocyte ratio >3.4 (HR 1.19; *p* = 0.033), and baseline ALP ≥230 U/L (HR, 1.06; *p* = 0.044).

Paganelli et al^25^ also explored this in a multinational expanded access program, analyzing OS by the number of ^223^Ra doses received. Patients who received ≤4 doses had a median survival of 6.3 months, while those who received >4 doses had a median survival of not reached. Administration of >4 doses was associated with lower baseline ECOG PS score (0–1), subjective improvements in pain, higher baseline Hb ≥100 g/L, and lower baseline PSA level <141 µg/L.

Dizdarevic et al^26^ found the median OS for 57 patients with mCRPC who received ^223^Ra for 6 doses was 13.3 months among patients with normal baseline ALP versus 7.4 months for those with elevated baseline ALP; *p* = 0.01. Patients who achieved ≥30% ALP reduction during the treatment course had better median OS compared to non‐responders (12.1 vs. 3.8 months; *p* = 0.01).

Our prognostic model showed an acceptable level of performance to define the prognostic groups, which is similar to that proposed by Halabi et al.^27^ They proposed an updated mCRPC model using CALGB–90401 training set, which included opioid analgesic use, LDH > 1x the upper limit of normal, disease site, ECOG PS, albumin, Hb, PSA, and ALP.

Previous attempts to create a prognostic model for mCRPC patients who received ^223^Ra produced a similar AUC to our study. Frantellizzi et al^28^ studied retrospectively 92 patients with mCRPC and symptomatic bone metastases who were treated with ^223^Ra. On UVA, prognostic baseline variables included body mass index, ECOG PS, Hb, and ALP values were associated with OS. However, after MVA, only ECOG PS and Hb were strongly associated with OS. In a far more complicated model, those authors tested a high number of variable combinations and discovered an optimal prognostic score. The model was achieved by combining three prognostic scores (3‐PS): ECOG PS, PSA ≥ 20 µg/L, and Hb < 120 g/L and produced an AUC 0.784; *p* < 0.001. They reported zero, one, and two points for baseline ECOG PS (0,1, ≥ 2), respectively, one point for baseline PSA ≥20 µg/L, and one point for baseline Hb < 12 g/dL. In a subgroup analysis of survival based on 3‐PS, cohort stratification showed median OS >31, 11, 9, and 4 months for the scores 0–1, 2, 3, and 4, respectively. Unlike our model, which clearly describes very distinct groups, this model has two reasonably similar groups with an OS only 2 months apart for those who scored 2 or 3 on the 3‐PS model.

The small number of patients included in the analysis limits the robustness of the findings. Our findings must be confirmed in a larger number of patients in an independent dataset. Furthermore, the variables identified by our model are not specific to ^223^Ra, and so the biologic rationale for the impact of these variables on survival with ^223^Ra is unclear.

In conclusion, pre‐treatment ALB, ALP, ECOG PS, and PSA, have significant correlations with OS and provide prognostic information that could be used to select patients who are most or least likely to benefit from ^223^Ra. Our model is simple, sensitive, and defines very distinct prognostic groups. The use of an independent dataset for validation should be promoted. Patients who received >4 doses of ^223^Ra, or systemic treatment after ^223^Ra or who have any PSA response during ^223^Ra therapy had better survival outcomes. The presence of any hematological toxicities after completion of ^223^Ra therapy was associated with poor survival outcomes.

## CONFLICT OF INTEREST

**Marco A.J. Iafolla** has received funding from Novartis (advisory board); Bayer, Canadian Urologic Association, Compass MD, Ipsen, Merck, Save Your Skin honorarium (Honoraria); and Astellas Pharma Global Development Inc., AstraZeneca, Bristol‐Myers Squibb (clinical trials). **Adrian G**. **Sacher** has received funding from AstraZeneca, Bayer (Consulting, Advisory Boards) and AstraZeneca, Merck, Genentech Roche, Bayer (Honoraria &Sponsored CME/Symposia). **Di M. Jiang** has received funding from Bayer (Honoraria). **Alexandre R**. **Zlotta** has received funding from Sanofi, Ferring, Janssen (Advisory Board). **Aaron R. Hansen** has received funding from Genentech/Roche, Merck, GSK, Bristol Myers Squibb, Novartis, Boston Biomedical, Boehringer Ingelheim, AstraZeneca, Medimmune (Advisory/Consulting/Research). All remaining authors have declared no conflict of interest.

## AUTHOR CONTRIBUTIONS

**Esmail M. Al‐Ezzi:** Conceptualization, data curation, formal analyses, methodology, investigation, software, writing––original draft, and writing––review and editing. **Husam A. Alqaisi:** Data curation, software, writing––original draft, and writing––review and editing **Marco A.J. Iafolla:** Conceptualization, data curation, methodology, and writing––original draft. **Lisa Wang:** Data curation, formal analyses, methodology, and writing––original draft. **Srikala S. Sridhar:** Conceptualization, resources, and writing––review and editing. **Adrian G**. **Sacher:** Conceptualization, resources, and writing––review and editing. **Nazanin Fallah‐Rad:** Conceptualization, resources, and writing––review and editing. **Di M. Jiang:** Resources, writing––original draft, and writing––review and editing. **Geoffrey A. Watson:** Conceptualization, resources, and writing––review and editing. **Charles N. Catton:** Conceptualization, resources, and writing––review and editing. **Padraig R**. **Warde:** Conceptualization, resources, and writing––review and editing. **Rob J. Hamilton:** Conceptualization, resources, and writing––review and editing. **Neil E**. **Fleshner:** Conceptualization, resources, and writing––review and editing. **Alexandre R**. **Zlotta:** Conceptualization, resources, and writing––review and editing. **Aaron R. Hansen:** Conceptualization, formal analyses, methodology, investigation, project administration, resources, software, supervision, writing––original draft, and writing––review and editing.

## ETHICAL APPROVAL STATEMENT

This study was conducted in accordance with good clinical practice, with the study protocol, the ethical principles stated in the declaration of Helsinki and the applicable laws and regulations. The study received ethics approval from the University Health Network institutional review board (REB #18‐5545).

## Supporting information

Appendix S1

## Data Availability

The data that support the findings of this study are available on request from the corresponding author. The data are not publicly available due to privacy or ethical restrictions.
