# Supplementary material for: Clinicopathologic factors that influence prognosis and survival outcomes in men with metastatic castration‐resistant prostate cancer treated with Radium‐223
Source: Cancer Med. 2021 Jul 13;10(17):5775–82. doi: 10.1002/cam4.4125 (PMC8419779; doi:10.1002/cam4.4125)
Supplement: Supplementary file 1 — Appendix S1 [file CAM4-10-5775-s001.docx]

**Supplementary file1**

**Case application of the study prognostic model**

**Case 1:**


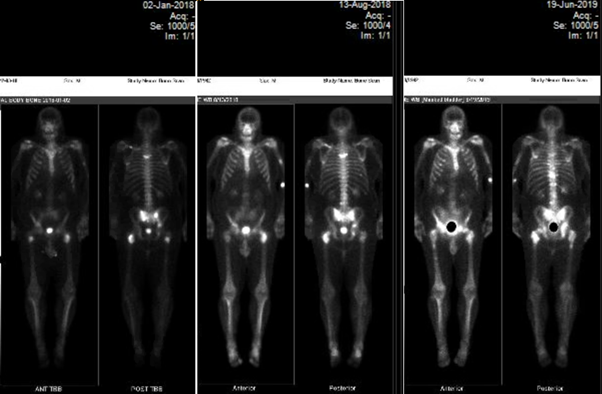


78-year-old male who diagnosed in 2013 with de novo metastatic prostate cancer
with a PSA 600 at presentation. At that time, CT scan showed disease involving the prostate, pelvic lymph nodes and bone. He has responded very well to hormonal therapy treatment with the CASODEX and LUPRON injections with an excellent biochemical and radiological response. Upon progression he had been started on ENZALUTAMIDE for biochemical recurrence in October 2015 with good response until February 2018. He progressed in the bone with multifocal disease T4-5 vertebra, the sacrum, ilium bilaterally, both proximal femora, and the right mid femoral shaft (see the above bone scan). CT scan did not show distant visceral metastasis. He started ^223^Ra for six cycles between March 2018 and August 2018. His Baseline clinical and laboratory test pre-Radium, ECOG 1, PSA 58 ug/L, ALK 165 U/L, ALB 37 g/L. His PSA during Radium treatment dropped from 58 to 39 ug/L. His staging CT and bone scan post radium showed stable disease findings. He developed disease progression biochemical and radiological in June 2019. He was not fit for systematic chemotherapy and was treated with multiple palliative radiotherapy and supportive treatment. He died in April 2020 with overall survival after ^223^Ra 25 months. Based on our prognostic model he was in good risk category with one point (ALK >150 U/L) and good PSA response during ^223^Ra.

**Case 2:**


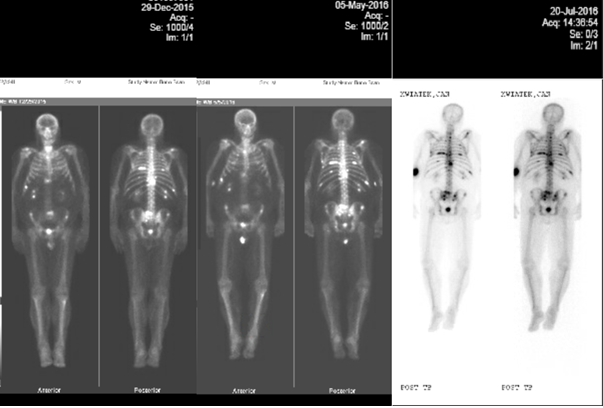


80-year-old male who diagnosed with prostate cancer, cT2a, Gleason 6, PSA 10.36, in 2006. He had a radical prostatectomy with pathology confirming a pT3a N0, Gleason 3 + 4 equals 7, with extra prostatic extension, and positive margins. Postoperative PSA is 0.07, April 2006. Subsequently He developed disease progression in the pelvic lymph nodes. He received ELIGARD for many years, BICALUTAMIDE was added on October 26, 2012, for rising PSA. PSA in September 2012 was 4.97. Restaging bone scan in October 2012 reported new activity right base of skull and CT head and facial bones in November 2012 reported a lesion in the right occipital condyle metastasis. He received palliative radiotherapy to the skull base. He developed disease progression in November 2013 and started on ENZALUTAMIDE until progression. This was followed by second line chemotherapy DOCETAXEL for ten cycles, between January and August 2015. Upon progression, he commenced on ABIRATERONE between December 2015 and March 2016 with progression. He started ^223^Ra in March 2016. His pre-Radium clinical and laboratory tests: ECOG 1, PSA 91 ug/L, ALK 206 U/L, ALB 34 g/L. His baseline bone scan (see the above bone scan) metastatic lesions in thoracolumbar spine, bilateral ribs, skull, and pelvis bilaterally. He finished six cycles of ^223^Ra. His PSA increased during radium treatment from 91 ug/L to 212 ug/L. His bone scan post radium 223 showed no interval changes. His condition deteriorated due to pathological fracture at his spine and became bedridden and died in October 2016. His overall survival after ^223^Ra 223, 8 months. Based on our prognostic model he is in poor risk category with three positive points (PSA>80 ug/L, ALK >150 U/L, ALB <35 g/L).
